# Supplementary material for: Learning curve and surgical outcome of robotic assisted colorectal surgery with ERAS program
Source: Sci Rep. 2022 Nov 29;12:20566. doi: 10.1038/s41598-022-24665-w (PMC9709162; doi:10.1038/s41598-022-24665-w)
Supplement: Supplementary file 1 — Supplementary Information 1. [file 41598_2022_24665_MOESM1_ESM.zip › LSC Raw data-Ñ[▒K/139/P_ñw╝╨░O▒Kñσ.pdf]

[redacted]

Pathologic diagnosis:
1. Ascending colon, ileum, and appendix, laparoscopic right hemicolectomy --- Status post polypectomy, no residual tumor is seen. The appendix, ileocecal valve, bilateral cut ends, vascular cut end, and mesocolic lymph nodes (0/29) are free of tumor.
2. Peritoneum of uterus, resection --- Unremarkable smooth muscle tissue and inflammatory exudate.

Gross description:
The specimen consists of a segment of colon, 16 cm in length, and 7 cm in proximal circumference, 6.5 cm in distal cut end circumference, and a segment of terminal ileum, 7 cm in length, and 4.5 cm in cut end circumference. An unremarkable appendix, 6 cm, is noted. No tumor is seen grossly. There is a clip labeled the previous polypectomy site, 8 cm to the distal cut end. The appendix, ileocecal valve, and both cut ends are free of tumor. Representative part for section: A) proximal cut end B) distal cut end C) ileocecal valve D) appendix E-H) mucosal of the clip site J) vascular cut end K) specimen 2 L-P) lymph nodes.
#T-59420_2 #M-09413_2 0 1105 SP0000
#T-C4560_2 #M-09183_2 0 1105 000000
#T-59200_2 #M-09183_2 0 1105 000000

已電子簽章

敬告：本單僅供醫師參考使用，不具任何申請效力！！
請病患妥為保管，若不慎遺失需自負個人資料外洩責任！
